# Adaptive Immunity Alters Distinct Host Feeding Pathways during Nematode Induced Inflammation, a Novel Mechanism in Parasite Expulsion

**DOI:** 10.1371/journal.ppat.1003122

**Published:** 2013-01-17

**Authors:** John J. Worthington, Linda C. Samuelson, Richard K. Grencis, John T. McLaughlin

**Affiliations:** 1 Manchester Immunology Group, Faculty of Life Sciences, University of Manchester, Manchester, United Kingdom; 2 Department of Molecular and Integrative Physiology, University of Michigan Medical School, Ann Arbor, Michigan, United States of America; 3 School of Translational Medicine, School of Medicine, University of Manchester, Manchester, United Kingdom; Cornell University, James A. Baker Institute of Animal Health, United States of America

## Abstract

Gastrointestinal infection is often associated with hypophagia and weight loss; however, the precise mechanisms governing these responses remain poorly defined. Furthermore, the possibility that alterations in feeding during infection may be beneficial to the host requires further study. We used the nematode *Trichinella spiralis*, which transiently inhabits the small intestine before migrating to skeletal muscle, as a biphasic model of infection to determine the cellular and molecular pathways controlling feeding during enteric and peripheral inflammation. Through the infection of genetically modified mice lacking cholecystokinin, Tumor necrosis factor α receptors and T and B-cells, we observed a biphasic hypophagic response to infection resulting from two separate immune-driven mechanisms. The enteroendocrine I-cell derived hormone cholecystokinin is an essential mediator of initial hypophagia and is induced by CD4+ T-cells during enteritis. In contrast, the second hypophagic response is extra-intestinal and due to the anorectic effects of TNFα during peripheral infection of the muscle. Moreover, via maintaining naive levels of the adipose secreted hormone leptin throughout infection we demonstrate a novel feedback loop in the immunoendocrine axis. Immune driven I-cell hyperplasia and resultant weight loss leads to a reduction in the inflammatory adipokine leptin, which in turn heightens protective immunity during infection. These results characterize specific immune mediated mechanisms which reduce feeding during intestinal or peripheral inflammation. Importantly, the molecular mediators of each phase are entirely separate. The data also introduce the first evidence that I-cell hyperplasia is an adaptively driven immune response that directly impinges on the outcome to infection.

## Author Summary

Infection with intestinal parasites often results in a period of reduced appetite which can result in weight loss; however the factors which control these feeding alterations and the reason why they occur is unknown. We used the nematode parasite *Trichinella spiralis*, which during its life cycle causes intestinal and muscular inflammation, as a mouse infection model to study the factors which alter feeding during infection. We found that the mouse immune response to the parasite was driving two periods of reduced feeding by two distinct immune mediators during the intestinal and muscular periods of infection. Interestingly, the immune system was utilizing a hormone which usually terminates feeding during our daily meals to cause a reduction in weight and fat deposits. Furthermore, we found that a reduction in these fat deposits and their associated hormones actually helped the mouse expel the parasite from the intestine. Hence the immune driven weight loss was actually beneficial to the mouse's ability to resolve an infection. Our study provides novel insights into how the immune system interacts with feeding pathways during intestinal inflammation and may help us design new strategies for helping people with parasitic infections of the gut.

## Introduction

Intestinal inflammation is commonly associated with reduced feeding (hypophagia) and weight loss [1], [2], yet the mechanisms and underlying principles of these responses is unknown. Infection with the intestinal parasites *Ascaris suum* and *Trichostrongylus colubriformis* results in hypophagia that is coupled with an increase in cholecystokinin (CCK) released from I-cells [3], [4]; a subset of intestinal epithelial enteroendocrine cells (EECs). Despite only comprising 1% of the epithelium, EECs collectively form the largest mammalian endocrine system. Regulatory peptides and amines are released from EECs in response to luminal nutrients [5] and these peptides signal via vagal afferent fibers to feeding control centers in the brain. These EEC signals in concert with leptin, produced from adipose tissues indicating levels of fat deposits, ultimately control our daily short-term feeding patterns. However, the true biological function and molecular mechanisms that orchestrate the pathways driving hypophagia and weight loss during inflammation have not been addressed.

The nematode *Trichinella spiralis* produces a well characterized CD4+ T-cell, Th2 driven transient inflammation in the small intestine culminating in worm expulsion via a mast cell dependent process [6]. Recently we have observed a hypophagic response during the Th2 driven enteritis induced by *T. spiralis* infection [7]. However, the full mechanisms controlling hypophagia during enteritis and the precise effects reduced feeding have on immunity to intestinal infection require further elucidation. *T. spiralis* is experimentally highly attractive since the enteritis fully resolves, but is closely followed by a peripheral inflammatory phase characterized by skeletal muscle invasion and myositis as part of the parasite's life cycle.

Here, we demonstrate that this two step inflammatory process following *T. spiralis* infection is mirrored by a biphasic hypophagic response, and mediated by two separate adaptive immune driven mechanisms. We have characterized these two phases using genetically modified mice lacking functional CCK or adaptive immunity and demonstrated that CD4+ T-cells drive I-cell hyperplasia and the resulting CCK is an essential mediator of the initial hypophagia observed during enteritis. Conversely the second phase of hypophagia during skeletal peripheral myositis is CCK-independent but mediated by the anorectic actions of TNFα signaling. Furthermore, we demonstrate for the first time that this immune-EEC driven alteration in feeding also contributes a protective role during gastrointestinal infection. The hypophagia and resulting weight loss causes a reduction in fat secreted leptin, and the reduction in this hormone, which also acts as an inflammatory adipokine, augments the protective Th2 immune response aiding parasite expulsion. These results highlight the importance of the immunoendocrine axis in the gut during infection induced immunity and provide a biological function and associated mechanism for commonly associated infection induced weight loss. These data have wide-acting implications for the biology of gut infection and inflammation, and may inform new leptin-derived therapeutic strategies. Furthermore, the *T. spiralis* infected mouse presents a novel preclinical platform to study the biological mechanisms affecting food intake in inflammatory disorders, and has the unique potential to experimentally dissociate gastrointestinal from peripheral signals in an individual model.

## Results

### *T. spiralis* induced enteritis and myositis induces a biphasic hypophagic response, for which CCK is crucial for the initial period during enteritis

**Figure 1:**
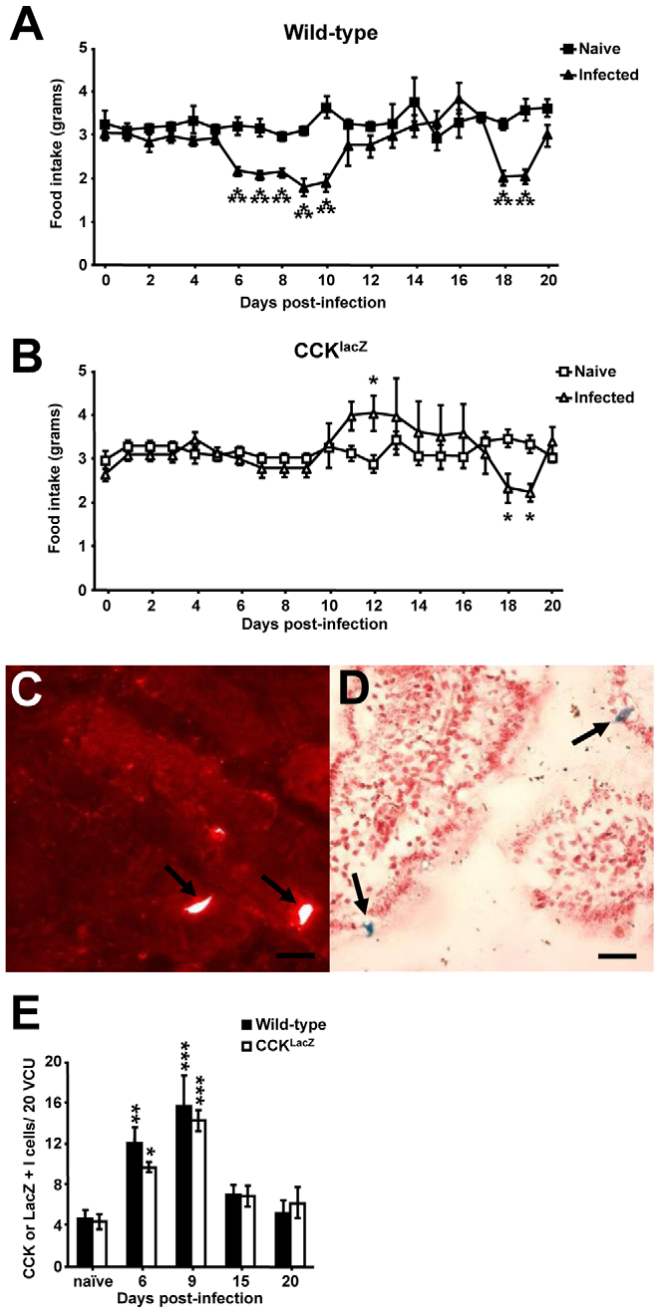
*T. spiralis* induced enteritis and peripheral myositis produces a CCK dependent and independent bi-phasic hypophagia. Food intake of naïve and infected wild-type (*A*) and CCK^lacZ^ (*B*) mice, derived via weighing chow daily. Representative CCK (*C*) and lacZ (*D*) I-cell staining from day 9 p.i. wild-type and CCK^lacZ^ duodenum respectively. Black arrows indicate I-cells. Black bar = 100 µm. (*E*) Number of CCK/LacZ positive I-cells/20 VCU in wild-type and CCK^lacZ^ mice as determined from immunohistochemistry or immunofluorescence respectively. Data (n = 8–10 mice/group) from two independent experiments. *, P<0.05; **, P<0.01; or ***, P<0.005 between naïve and infected groups, error bars represent SE of means.

Proximal enteritis induced by *T. spiralis* has been associated with a period of hypophagia and an increase in CCK and serotonin secreting EECs [7]. Here, mice were examined for alterations in feeding during *T. spiralis* induced inflammation. Interestingly, a biphasic response in feeding was observed following infection (Fig. 1A). Mice became hypophagic from days 6–10 post infection (p.i.), during the transient period of *T. spiralis* induced enteritis, and we observed a significant increase in CCK positive I-cells in wild-types (Fig. 1C and E) mirroring hypophagia at days 6 and 9 p.i. Feeding then returned to baseline levels until undergoing a second period of hypophagia from day 18–19 p.i. The secondary period of hypophagia occurs during the period of muscle invasion and peripheral myositis, caused when larvae form the “nurse cell” in which the parasite resides. To further investigate the biological mediators of the hypophagic responses the two phases were mechanistically explored using a panel of genetically modified mouse strains.

CCK^lacZ^ mice, which do not express or secrete CCK peptide due to a knock in of a LacZ cassette [8], were infected with *T. spiralis* and their food intake monitored. Strikingly, the initial period of hypophagia was completely absent in infected CCK^lacZ^ mice (Fig. 1B). LacZ positive I-cell hyperplasia was indistinguishable from that of the “natural” I-cell response in wild-type mice (Fig. 1D and E). However, the absence of CCK during this I-cell hyperplasia resulted in the complete absence of initial hypophagia in CCK^lacZ^ mice, despite comparable enteritis (Fig. S1). Even with the absence of CCK and initial hypophagia, CCK^lacZ^ mice still exhibited secondary hypophagia from day 18–19 p.i. and this was comparable to infected wild-type mice (Fig. 1B). The second period of hypophagia occurred in wild-type and CCK^lacZ^ mice despite the resolution of enteritis (Fig. S1) and transpires during the period of larvae encysting within skeletal muscle, representing an extraintestinal inflammatory response to the same biological agent. The lack of I-cell hyperplasia in wild-type mice at day 20 p.i. (Fig. 1E) and the presence of the second phase of hypophagia in CCK^lacZ^ mice (Fig. 1B) confirm this extraintestinal period of hypophagia during myositis as CCK independent.

Taken together, these data demonstrate a biphasic hypophagia correlating to *T. spiralis* induced enteritis and peripheral myositis, respectively. Furthermore, increased I-cell function, through the release of CCK, is essential for the initial hypophagia during enteritis, but not the secondary episode during peripheral myositis.

### The adaptive immune system drives biphasic hypophagia during *T. spiralis* induced enteritis and peripheral myositis

**Figure 2:**
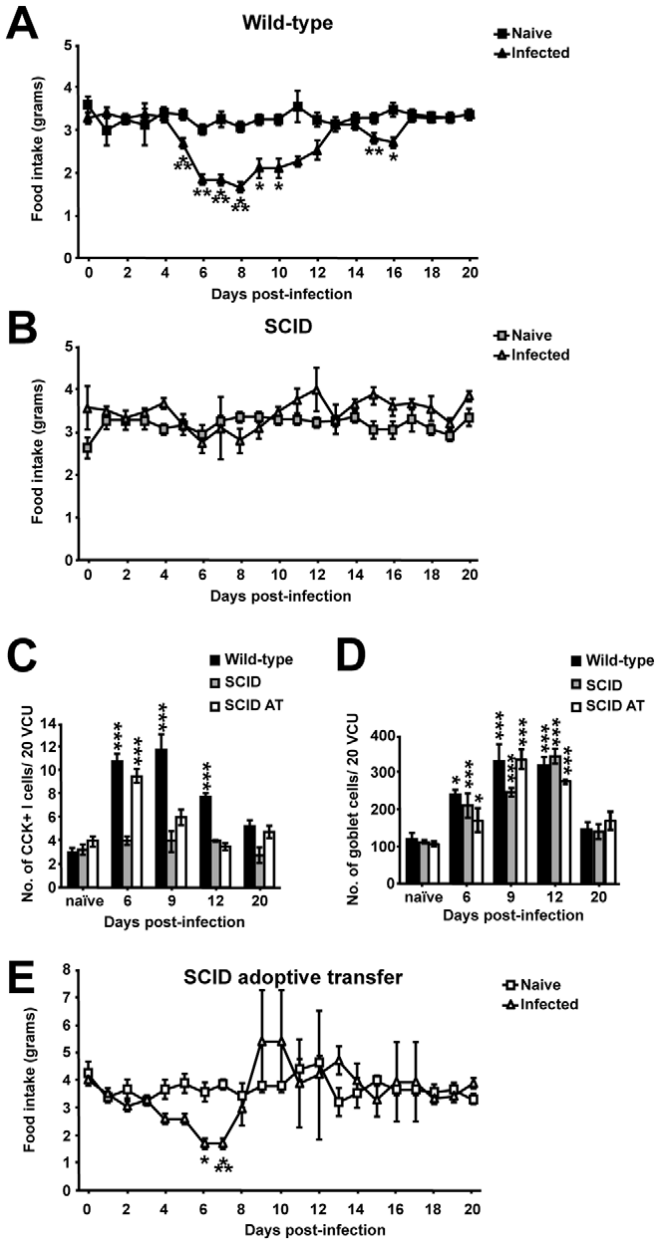
Adoptive transfer of CD4+ T-cells to SCID mice restores hypophagia during enteritis. Food intake of naïve and infected wild-type (*A*), SCID (*B*) and adoptively transferred SCID mice (*E*), derived via weighing chow daily. Number of CCK positive I-cells in wild-type, SCID and adoptively transferred SCID mice (*C*) and number of goblet cells in wild-type SCID and adoptively transferred SCID mice (*D*); cells/20 VCU accessed via immunohistochemistry and periodic acid-Schiff's histology staining respectively. Data (n = 4 mice per group) from 2 independent experiments. *, P<0.05; **, P<0.01; or ***, P<0.005 between naïve and infected groups, error bars represent SE of means.

As EEC hyperplasia during inflammation has been previously linked to T-lymphocytes [7], [9], the biphasic hypophagia generated by *T. spiralis* was examined in severe combined immunodeficient (SCID) mice, which lack B and T-cells. SCID mice demonstrated a complete absence of initial hypophagia during *T. spiralis* induced enteritis and secondary hypophagia during peripheral myositis (Fig. 2A and B). The lack of hypophagia was mirrored by a complete lack of I-cell hyperplasia in parasitized SCID mice (Fig. 2C). This absence of hyperplasia was not seen in all epithelial secretory cells as, concurrent with previous findings [10], indistinguishable goblet cell hyperplasia occurred in infected SCID, adoptively transferred SCID and wild-type animals (Fig. 2D).

CD4+ T-cells play a key role in the resolution of *T. spiralis* infection [11], so to assess if CD4+ T-cells could restore I-cell hyperplasia and hypophagia in SCID mice, CD4+ T-cells (>90% purity; Fig. S2A), were adoptively transferred into SCID recipients before infection. Successful reconstitution was evident from CD4+ splenocytes present post-transfer and via successful worm expulsion kinetics (Fig. S2B and C).The adoptive transfer of CD4+ T-cells into SCID mice restored I-cell hyperplasia (Fig. 2C) and initial hypophagia during *T. spiralis* induced enteritis. Recipient mice began to eat less from day 4 p.i., with significant hypophagia at days 6 and 7 (Fig. 2E). This hypophagia was not a direct result of cell transfer alone, as uninfected reconstituted mice displayed no hypophagia (Fig. 2E). Interestingly, the adoptive transfer did not restore the secondary period of hypophagia during the peripheral inflammation induced by *T. spiralis* (Fig. 2A).

Collectively, these data confirm that the biphasic alterations in feeding behavior during *T. spiralis* induced gastrointestinal and peripheral inflammation is mediated by the adaptive immune system. Furthermore, CD4+ T-cells are identified as the key initiator in I-cell hyperplasia and resulting CCK driven hypophagia during *T. spiralis* induced enteritis. However, the adoptive transfer of functional CD4+ T-cells did not restore the second phase of hypophagia occurring during nurse cell formation-induced myositis. Therefore CD4+ T-cells are not sufficient for this secondary hypophagic period, during *T. spiralis* induced myositis.

### The secondary period of hypophagia seen during *T. spiralis* induced peripheral inflammation is caused by the cachectic cytokine TNFα

**Figure 3:**
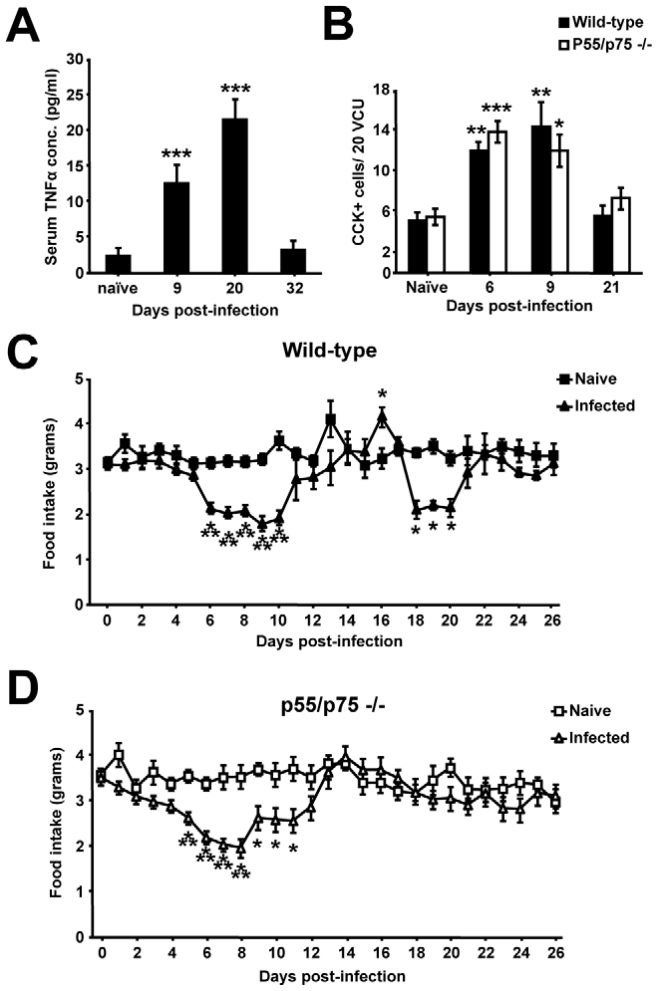
Secondary hypophagia during *T. spiralis* induced peripheral inflammation is absent in p55/p75 −/− mice. (*A*) TNFα serum levels during infection in wild-type mice determined via cytometric bead array (*B*) Number of CCK positive cells/20 VCU, as determined from immunohistochemistry in wild-type and p55/p75 −/− mice. Food intake of naïve and infected wild-type (*C*) and p55/p75 −/− (*D*) mice, derived via weighing chow daily. Data (n = 7–16 mice per group) are from three independent experiments. *, P<0.05; **, P<0.01; or ***, P<0.005 between naïve and infected groups, error bars represent SE of means.

We next sought to investigate which factors of the adaptive immune response were responsible for the second phase of hypophagia seen during peripheral inflammation induced during the period of nurse cell formation. Both CD4+ and CD8+ T-cells are present during parasite encystation [12] and many apoptotic factors, including TNFα, are detected during nurse cell formation [13]. Interestingly, TNFα is associated with cachexia in parasite infections [14], [15]. Consequently, we examined serum cytokine levels throughout *T. spiralis* infection. Indeed, TNFα was significantly increased in the serum of infected mice at the time of secondary hypophagia (Fig. 3A), comparable to levels known to directly cause cachexia in mouse infection models [16]. To test the function of increased TNFα during myositis we infected p55/p75−/− mice, which lack functional TNFα receptors, and assessed if TNFα was responsible for hypophagia during *T. spiralis* infection. Although initial I-cell hyperplasia and hypophagia during enteritis were present in infected p55/p75−/− mice (Fig. 3B, C and D), remarkably, infected p55/p75−/− mice displayed no period of secondary hypophagia (Fig. 3B and C). Therefore, although the initial CD4+ T-cell and CCK driven hypophagia during enteritis is independent of TNFα, a peripheral peak in TNFα during myositis is functionally responsible for the second phase of hypophagia, via the receptors p55 and/or p75, during *T. spiralis* induced peripheral inflammation.

### CD4+ T-cell mediated hypophagia results in an augmented protective immune response, through reduction in the pro-inflammatory adipokine leptin

Secretory cell hyperplasia during intestinal infection is known to be advantageous during infection. Various goblet and Paneth cell products have been show to have anti-parasitic affects [17], [18]. We therefore tested whether I-cell hyperplasia and hypophagia are simply by-products of a parallel switch towards this secretory lineage, or whether I-cell hyperplasia is in itself advantageous during infection. A link between hypophagia, weight loss and immunity is the adipokine leptin; produced mainly by adipose tissue it is a peripheral signal to the body of fat mass deposits but also acts as a pro-inflammatory Th1 cytokine [19]. Therefore reductions in leptin would be anticipated to occur following CCK induced hypophagia and consequent weight loss in this experimental model. Loss of leptin may consequently enhance Th2 immune responses which are protective during nematode infection.

**Figure 4:**
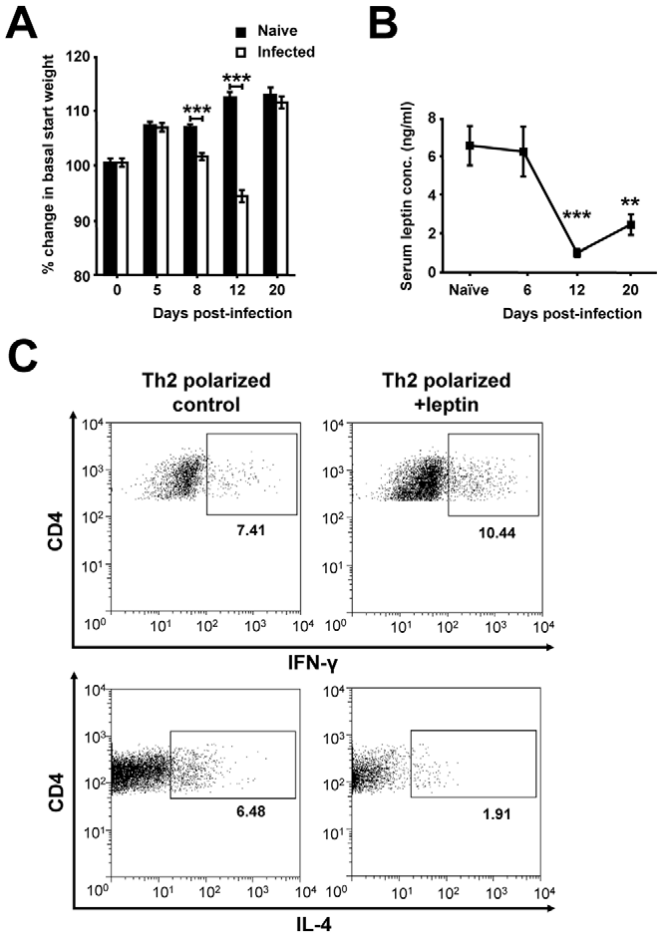
Hypophagia and weight loss during *T. spiralis* induced enteritis reduces the pro-inflammatory adipokine leptin. (*A*) Change in basal weight during infection. (*B*) Serum leptin levels during infection, determined via ELISA. (*C*) Representative CD4+ intracellular IFN-y and IL-4 flow cytometry plots during Th2 polarization in control and leptin treated mLN cells. Data (n = 4–8 mice per group) are from two independent experiments. *, P<0.05; **, P<0.01; or ***, P<0.005 between naïve and infected groups or for indicated comparisons, error bars represent SE of means.

**Figure 5:**
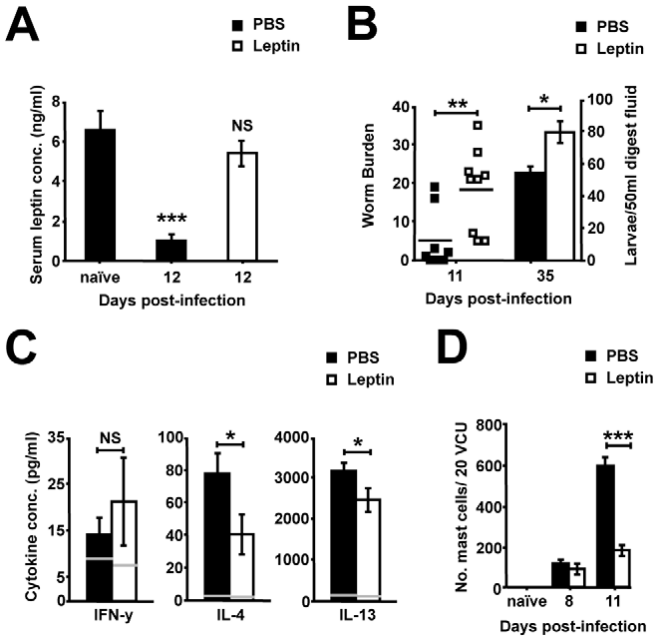
The maintenance of basal leptin levels during *T. spiralis* infection results in delayed parasite expulsion. (*A*) Serum leptin levels in PBS and leptin treated mice, determined via ELISA. (*B*) Adult and larval worm burdens in infected mice. (*C*) IFN-y, IL-4 and IL-13 cytokine levels from Ag-specific re-stimulation of day 8 p.i. mLN cells from PBS and leptin treated mice, grey lines represent naïve levels; determined via cytometric bead array. (*D*) Mast cells/20 VCU in PBS and leptin treated mice; accessed via toludine blue staining. Data (n = 9 mice per group) are from two independent experiments. *, P<0.05; **, P<0.01; or ***, P<0.005 between naïve and infected groups or for indicated comparisons, error bars represent SE of means.

CD4+ T-cell mediated I-cell driven hypophagia during enteritis was seen to result in significant weight loss at days 8 and 12 p.i., accompanied by a visible reduction in abdominal fat pads, whereas the brief TNFα driven secondary hypophagia produced no significant alteration in weight at day 20 p.i. (Fig. 4a). This weight loss was correlated with a reduction in serum leptin levels from day 6 p.i. (Fig. 4B). To determine whether alterations in leptin could influence a protective Th2 driven intestinal I immune response, mesenteric lymph node (mLN) cells were polarized towards a Th2 phenotype in the presence or absence of leptin. The addition of leptin resulted in a significant increase in the amount of intracellular pro-inflammatory IFN-y detectable in CD4+ T-cells, as well as a significant reduction in the protective Th2 cytokine IL-4 (Fig. 4C). To assess if the reduction in leptin during *T. spiralis* induced enteritis enhances immunity to infection, leptin levels were maintained at basal levels during hypophagia via recombinant leptin injection (Fig. 5A). Strikingly, the restoration of basal leptin levels resulted in delayed expulsion of adult worms and a corresponding increase in nurse cell encystation (Fig. 5B). Although no increase in IFN-y levels was seen in re-stimulated mLNs of leptin treated mice, a significant decrease in both Th2 cytokines IL-4 and IL-13 was seen at day 8 p.i (Fig. 5C). A key Th2 driven expulsion mechanism of *T. spiralis* is mastocytosis and this was seen to be significantly reduced upon the restoration of basal leptin levels analogous to delayed adult worm expulsion (Fig. 5D). Taken together this suggests that CD4+ T-cells drive a cascade in which I-cell hyperplasia produces hypophagia and weight loss, lowering pro-inflammatory leptin levels which feed back to influence the protective Th2 immune response, augmenting mastocytosis and allowing parasite expulsion.

## Discussion

During enteritis food intake is often significantly reduced, perhaps serving to inhibit consumption of contaminated food or to prevent further gut injury. EECs are implicated in this response: elevated I-cell produced CCK and hypophagia have been demonstrated during *Ascaris suum* and *Trichostrongylus colubriformis* infection, leading to the hypothesis that CCK was responsible for inflammation induced alterations in feeding [3], [4] However, the true biological function and molecular mechanisms that orchestrate the pathways driving hypophagia and weight loss during inflammation have not been addressed. The life cycle of *T. spiralis* has uniquely allowed us to investigate these questions during both intestinal and peripheral inflammation. Our data demonstrate that these separate inflammatory episodes are mirrored by a biphasic hypophagia driven by two independent immune mediated mechanisms. I-cell hyperplasia and CCK are essential for the initial hypophagia during enteritis, which is orchestrated by CD4+ T-cells. Importantly, we have also identified the second phase of hypophagia during the period of nurse cell formation to be mediated by a separate myositis-induced immune mechanism, fully dependent on the actions of TNFα.

Furthermore we show for the first time that immune driven weight loss during enteritis results in reduced levels of the Th1 adipokine leptin augmenting a protective Th2 response during infection. Intestinal inflammation is often associated with hypophagia and weight loss [1], [2] and we have now determined an important immune driven mechanism to explain why this is biologically functional. We have therefore identified a novel molecular pathway and can include I-cell hyperplasia and weight loss as an adaptively driven immune response which, through alterations in leptin, is beneficial during intestinal infection.

Our finding that *T. spiralis* infected mice lacking CCK (CCK^lacZ^) do not undergo initial hypophagia correlates with our own and other previous findings that a single treatment with the CCK_1_ receptor antagonist loxiglumide partially restores food intake in parasitized animals [7], [20]. This partial restoration now seems likely a result of the short half-life of loxiglumide as opposed to alternative satiety factors playing an essential role. The previous characterization of CCK^lacZ^ mice demonstrated normal food intake, fat absorption and mass compared to wild-types [21], [22]. This coupled with our own observations rules out any underlying defect in feeding of CCK^lacZ^ mice being responsible for the absence of hypophagia during infection. An alternative possibility is that the loss of CCK in brain neurons, rather than gut EECs, underpins the complete absence of hypophagia. However, the persistence of the second phase of hypophagia in CCK^lacZ^ mice and our previous findings involving loxiglumide [7] which does not cross the blood brain barrier, make I-cell hyperplasia and CCK-vagal interactions most likely to mediate gut-induced hypophagia.

*T. spiralis* infected SCID mice were seen to display I-cell hyperplasia and hypophagia only upon reconstitution of CD4+ T-cells. These data clearly indicate that CCK induced satiety, via vagal afferent fibers signaling to feeding control centres in the brain [5], is an inherent pathway that is utilized by the adaptive immune system to bring about hypophagia and weight loss. This finding corresponds to recent studies showing that CD4+ T-cells restore 5-HT cell hyperplasia in *Trichuris muris* infected SCID mice [23]. The precise mechanism by which CD4+ T-cells cause EEC hyperplasia during infection remains to be elucidated. EECs have been shown to possess functional TLRs [24] and IL-13 receptors are present on 5-HT cells [9]. However, it has previously been established that during *T. spiralis* infection in SCID mice NK cells produce ample levels of IL-13 to induce goblet cell hyperplasia [10] yet this IL-13 appears not sufficient to cause EEC hyperplasia. We also detected mRNA for both TNFα receptors p55 and p75 on EECs, yet mice genetically deficient in TNF receptor signaling demonstrated initial hypophagia and enteritis (Fig. S3A–C) arose independently of its actions.

As all gut epithelial subtypes are derived from pluripotent crypt stem cells [25], immune cell mediators may alter the transcription factors at the stem cell level leading to altered EEC hyperplasia during infection. Alternatively, analogous alterations in the post-stem cell, neurogenin+, EEC specific progenitor cells could alter EEC proliferation. BrdU labeling studies in experimentally-induced inflammation demonstrated EEC hyperplasia does occur at the level of the stem/progenitor cell rather than fully differentiated epithelial cells [26]. Indeed, the uncoupling of goblet and I cell hyperplasia seen here in infected SCID mice supports the hypothesis that neurogenin+ EEC precursors, rather than the stem cell itself, is targeted by an unknown CD4+ dependent mechanism during *T. spiralis* driven I-cell hyperplasia. Identifying which factors drive this CD4+ T-cell-stem/progenitor cell interaction is an exciting area for further study.

We have identified a novel and specific role for TNFα in hypophagia during *T. spiralis* induced peripheral inflammation. The absence of the second phase of hypophagia from p55/p75 −/− mice suggests that the drop in food intake during this extra-enteric inflammatory period is due to the anorexic effects of systemic TNFα or downstream targets. The significant peak of serum TNFα seen in mice correlated with the second period of hypophagia, occurring during nurse cell development and myositis, and strongly supports this notion. Indeed, we observed serum levels comparable to levels known to directly cause cachexia in mouse infection models [16]. Furthermore, TNFα is associated with cachexia in *Trypanosoma cruzi* infection [14] and during schistosomiasis [15]. There are numerous modes of action by which TNFα could cause anorexia [27]. Although central TNFα levels were not directly monitored, the 20 pg/ml serum TNFα measured during secondary hypophagia is below the levels required to induce anorexia by central administration [28]. It is therefore most likely that TNFα is acting on peripheral afferent nerves, as low level localized cytokine production can trigger afferent nerves without causing an increase in circulating cytokine levels [29]. It is also possible that myalgia and malaise may have contributed to reduced food intake: appetite per se cannot be measured in mice.

The observed systemic peak in TNFα occurs during the period of encystation of *T. spiralis* new born larvae in cells of the striated muscle. Encystations are likely to arise from day 4–10 post-infection with rapid growth of the parasite occurring over the following 20 days as terminally differentiated muscle cells re-enter the cell cycle and establish a niche for the parasite [12]. Early non-significant increases in systemic TNFα were seen as early as day 8 post-infection; day 18–21 post-infection may therefore indicate a “tipping point” in peripheral TNFα levels, where significant myositis breaches the threshold required to produce anorexia. TNFα has been shown to be involved in nurse cell formation [13], yet we observed no alteration in nurse cell development in p55/p75−/− mice that could alternatively explain the observations seen (Fig. S3C–E). The cellular source of TNFα remains to be elucidated. CD4+ and CD8+ T-cells are reported to be present during parasite encystation [12], as are macrophages interestingly peaking during hypophagia [30]. However, as we illustrate here, given the absence of secondary hypophagia in SCID mice reconstituted with CD4+ T-cells, where macrophages are present, the likelihood is that CD8+ T-cells may be the source of cachectic TNFα. Further studies are therefore required to ascertain the cellular source of TNFα which drives the hypophagia during *T. spiralis* induced myositis.

Immune mediated secretory cell hyperplasia during intestinal infection is advantageous as goblet and Paneth cell products have been show to have anti-parasitic affects [17], [18]. We therefore postulated whether I-cell hyperplasia and hypophagia are simply by-products of a parallel switch towards these beneficial secretory lineages or whether I-cell hyperplasia is in itself advantageous in nematode expulsion. Stimulation of the vagus nerve via nutritional release of CCK has also been shown to protect against hemorrhagic shock [31]. Therefore I-cell hyperplasia during nematode infection may represent a previously unidentified anti-inflammatory response. We therefore hypothesized that a reduction in weight as a result of I-cell induced hypophagia may alter the levels of the Th1 adipokine leptin [19]. A reduction in leptin could enhance the protective Th2 immune response to nematode infection. Indeed significant weight loss and reduced leptin levels did occur during *T. spiralis* induced hypophagia. Recent data on splenocytes demonstrated that leptin alters polarized CD4+ T-cells towards a Th1 phenotype via alterations in proliferation *in vitro* [32] and we demonstrated parallel results in mLN cells for the first time. Unfortunately CCK^lacZ^ mice have overall reduced basal levels of leptin [33] and were hence unsuitable to study the affect of reduced leptin on intestinal inflammation. We therefore maintained basal leptin levels in infected hypophagic mice and strikingly saw a significant reduction in Th2 cytokines and mastocytosis culminating in delayed worm expulsion. Interestingly mastocytosis was similar in both leptin reconstituted and wild-type mice at day 8 p.i. demonstrating that initially mastocytosis can establish, but without the I-cell driven reduction in Th1 polarizing leptin it is blunted later in infection. These results complement other recent studies in identifying the adipokine leptin as a molecule which can greatly influence the response to infection. Mice lacking the leptin receptor are highly susceptible to infection from protozoa [34], pneumonia [35] and Listeria [36] demonstrating how malnutrition can compromise Th1 driven immunity. However, our data demonstrate that brief alterations in leptin can benefit immunity in terms of Th2 driven resistance to infection. Indeed, a recent study has demonstrated that leptin receptor deficient mice are resistant to experimentally induced Th2-mediated colitis [32]. The precise action of leptin in our studies may be as a direct result of effects on CD4+ T-cell IL-4 production altering mast cell differentiation, proliferation and migration [37] or due to direct effects on mast cells which have recently been shown to express leptin receptors [38]. Leptin may also directly act on Th2 cytokine production itself as opposed to indirect alterations on Th1 cytokine production [32]. Further study is therefore required to address the leptin-mast cell axis which alters parasite expulsion in our model.

In conclusion, we have identified two separate immune mediated mechanisms of hypophagia during infection induced gastrointestinal and peripheral inflammation, which act via the distinct pathways of I-cell hyperplasia and TNFα cachexia. Furthermore, we demonstrate for the first time an immunoendocrine feedback loop, in which CD4+ T-cell driven weight loss via CCK reduces leptin levels which impinge on CD4+ T-cell driven effector mechanisms for gastrointestinal infection resolution. Our data elucidate inflammation and weight loss, not just as commonly associated phenomena, but highlights them as a novel immune driven mechanism in parasite expulsion. These data offer potential specific treatment targets to modulate feeding and immune function during inflammatory diseases of the intestine.

## Materials and Methods

### Ethics statement

Mice were housed in specific pathogen free conditions and experiments were carried out in accordance with the United Kingdom Home Office Scientific Procedures Act (1986) under Department for Environment, Food and Rural Affairs license.

### Animals

Male C57BL/6 and BALB/c mice were obtained from Harlan-Olac Ltd. CCK^lacZ^ mice have a LacZ cassette knocked into the CCK locus on a C57BL/6 background, so homozygote animals are CCK null but faithfully express LacZ in the I cell population [8]. TNFα receptor null p55/p75 −/− (C57BL/6 background) and severe combined immunodeficient mice (SCID, BALB/c background) were generated as previously described [8], [39].

### Parasites

The maintenance, infection and recovery of *T. spiralis* were carried out as previously described [40].

### Food intake and body weight

Mice were individually weighed on a daily basis. Food intake per mouse was derived by weighing the chow (B and K, Hull, UK) daily.

### Immunohistochemistry and histology

Proximal small intestine was fixed and stained and I-cells were enumerated using, CCK specific, L421 anti-proCCK as previously described [7]. For CCK^lacZ^ detection, transverse 12 µm sections of tissue were cut and fixed in 0.2% glutaraldehyde and stained with X-gal as previously described [8]. Mast or goblet cell sections were stained in toludine blue or Schiff's reagent, respectively. After mounting, positive cells were enumerated in 20 randomly selected villus crypt units (VCU) and results presented as mean number of positive cells/20 VCU (± s.e.).

### Adoptive transfer of CD4+ T-cells to SCID mice

Mesenteric lymph node (mLN) cells were prepared from day 7 p.i. BALB/c mice, in RPMI-1640, supplemented with 10% fetal calf serum, 100 µg/ml penicillin/streptomycin and 1 mM L-glutamine (complete media). CD4+ T-cells were isolated via negative selection using an isolation kit (Miltenyi Biotec). Evaluation of CD4+ purity was via flow cytometry. SCID mice received 4×10^6^ cells in P.B.S. via intraperitoneal (i.p.) injection 2 days before infection.

### Isolation and *in vitro* restimulation of mLN cells

mLN cells at 5×10^6^ cells/ml in complete media received 50 µg/ml of *T. spiralis* antigen (Ag). Supernatants were collected after 24 hrs and cytokines measured using a cytometric bead array kit (BD).

### Serum cytokine detection

Serum was obtained from blood at the time of sacrifice via centrifugation at 15000×g and cytokines measured using a cytometric bead array kit (BD).

### Leptin ELISA

Mouse leptin ELISA (Linco) was used to detect mouse serum leptin according to manufacturer's instructions.

### Restoration of basal leptin levels during hypophagia

During the period of significant hypophagia, mice were treated at 10 a.m. and 6 p.m. via an i.p. injection of recombinant leptin (R and D) at 0.5 µg/g of initial body weight or control vehicle PBS [41].

### *In vitro* Th2 polarization of mLN cells

2×10^6^/ml mLN cells were stimulated via 5 µg/ml αCD28, 3 µg/ml αCD3 (BD) and polarized via 50 ng/ml IL-4 (Peprotech), 50 µg/ml anti-IFN-γ with/without 500 ng/ml recombinant leptin. At 120 hrs 1 µg/ml Brefeldin A/1 µg/ml monensin (Sigma-Aldrich) for IFN-y/IL-4 staining was added for 4 hrs before blocking with anti-FcγR (BD). Cells were stained for CD4 (BD) for 30 mins at 4°C before fixing in FACS fix buffer (1% formaldehyde, 0.1% BSA and 0.05% NaN_3_ in PBS). Cells were permeabilised in 0.1% saponin (Sigma-Aldrich) and stained with biotinylated anti-IFN-γ/anti-IL-4 (BD) for 25 mins at RT. Controls were stained with isotype controls (BD). Biotinylated antibodies were detected by streptavidin APC conjugate (Caltag) at 1/200 in saponin for 25 minutes RT. Cells were analyzed on a FACScalibur using Flowjo.

### Statistics

Two experimental groups were compared using Student's t-test. Three or more groups were compared using the Kruskal-Wallis test, Dunn's multiple comparison post-test. A p value of ≤0.05 was considered statistically significant. *, P<.05; **, P<.01; or ***, P<.005 for indicated comparisons, error bars represent SE of means.

## Supporting Information

Figure S1 **Enteritis in Wild-type and CCK^lacZ^ mice is comparable during** ***T. spiralis*** **infection.** (*A*) Comparison of crypt and villus length in wild-type and CCK^lacZ^ mice at naïve, 6, 12 and 20 days p.i. mice, quantified using Image J software. (*B*) Number of goblet and (*C*) mast cells/20 VCU in wild-type and CCK^lacZ^ mice in naïve, 6, 12 and 20 days p.i.; accessed via periodic acid-Schiff's and toludine blue histology staining respectively. Data (n = 4–8) from 2 independent experiments. *, P<0.05; **, P<0.01 or ***, P<0.005 between naïve and infected groups, error bars represent SE of means.
(TIF)

Figure S2 **Conformation of adoptive transfer of CD4+ T-cells in SCID mice.** Flow cytometry plot of (A) MACS purified donor CD3/CD4+ T-cells from day 6 p.i. wild-type mice and (B) representative plot of reconstitution into SCID mice on day 8 recipient splenocytes. Numbers represent percentage of cells in gate of overall lymphocyte gated cells. (C) Worm burdens recovered from wild-type, SCID and SCID adoptively transferred mice at days 6 and 20 p.i. Data (n = 4 mice per group). *, P<0.05; **, P<0.01 or ***, P<0.005 for the indicated comparisons, error bars represent SE of means.
(TIF)

Figure S3 **Enteropathy and nurse cell formation during** ***T. spiralis*** **infection is not altered in p55/p75−/− mice.** (A) Comparison of crypt and villus length in wild-type and p55/p75−/− mice at naïve, 6 and 21 days p.i., quantified using Image J software. (B) Number of goblet and (C) mast cells/20 VCU in wild-type and p55/p75−/− mice in naïve, 6, 12 and 21 days p.i.; accessed via periodic acid-Schiff's and toludine blue histology staining respectively. (D) Number of nurse cells visible in field of view in the diaphragm and rectus femoris of infected mice at 21 and 32 days p.i. (E) Cellular infiltration of eosinophils of nurse cells in the diaphragm and rectus femoris of infected mice at days 21 and 32 p.i. (F) Representative haematoxylin and eosin stained images from (E) Black bar = 100 µm. *A–D* determined via haematoxylin and eosin histological staining. A–F values represent the means ± SE (n = 4) from 2 independent experiments. *, P<0.05; **, P<0.01 or ***, P<0.005 between naïve and infected groups, error bars represent SE of means.
(TIF)
