# Supplementary figures and images for: Adaptive Immunity Alters Distinct Host Feeding Pathways during Nematode Induced Inflammation, a Novel Mechanism in Parasite Expulsion
Source: PLoS Pathog. 2013 Jan 17;9(1):e1003122. doi: 10.1371/journal.ppat.1003122 (PMC3547840; doi:10.1371/journal.ppat.1003122)

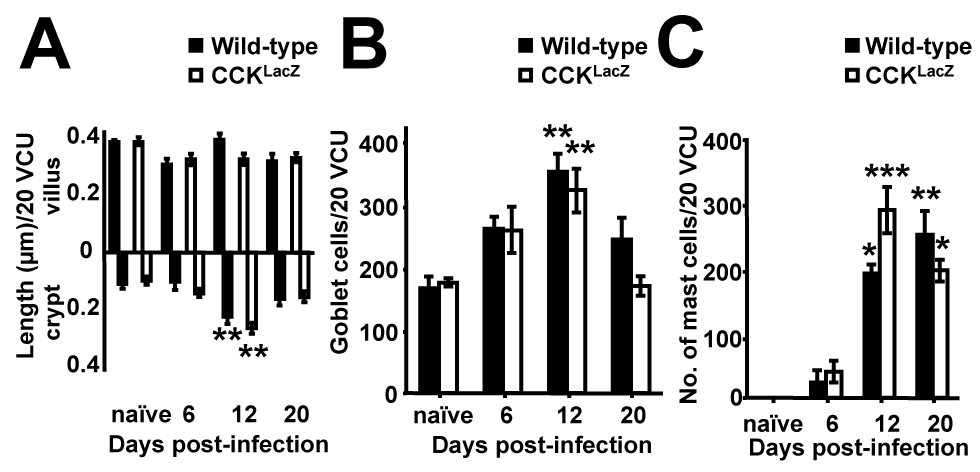

Supplement: Figure S1 — Enteritis in Wild-type and CCKlacZ mice is comparable during T. spiralis infection. (A) Comparison of crypt and villus length in wild-type and CCKlacZ mice at naïve, 6, 12 and 20 days p.i. mice, quantified using Image J software. (B) Number of goblet and (C) mast cells/20 VCU in wild-type and CCKlacZ mice in naïve, 6, 12 and 20 days p.i.; accessed via periodic acid-Schiff's and toludine blue histology staining respectively. Data (n = 4–8) from 2 independent experiments. *, P<0.05; **, P<0.01 or ***, P<0.005 between naïve and infected groups, error bars represent SE of means. (TIF) [file ppat.1003122.s001.tif]

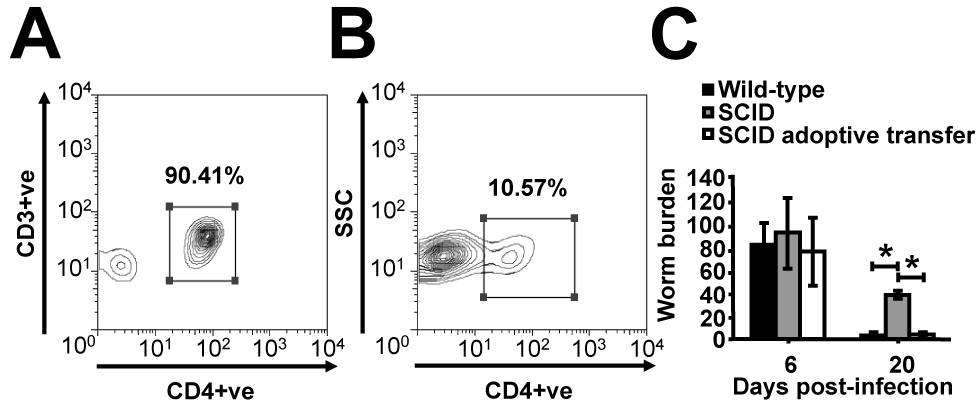

Supplement: Figure S2 — Conformation of adoptive transfer of CD4+ T-cells in SCID mice. Flow cytometry plot of (A) MACS purified donor CD3/CD4+ T-cells from day 6 p.i. wild-type mice and (B) representative plot of reconstitution into SCID mice on day 8 recipient splenocytes. Numbers represent percentage of cells in gate of overall lymphocyte gated cells. (C) Worm burdens recovered from wild-type, SCID and SCID adoptively transferred mice at days 6 and 20 p.i. Data (n = 4 mice per group). *, P<0.05; **, P<0.01 or ***, P<0.005 for the indicated comparisons, error bars represent SE of means. (TIF) [file ppat.1003122.s002.tif]

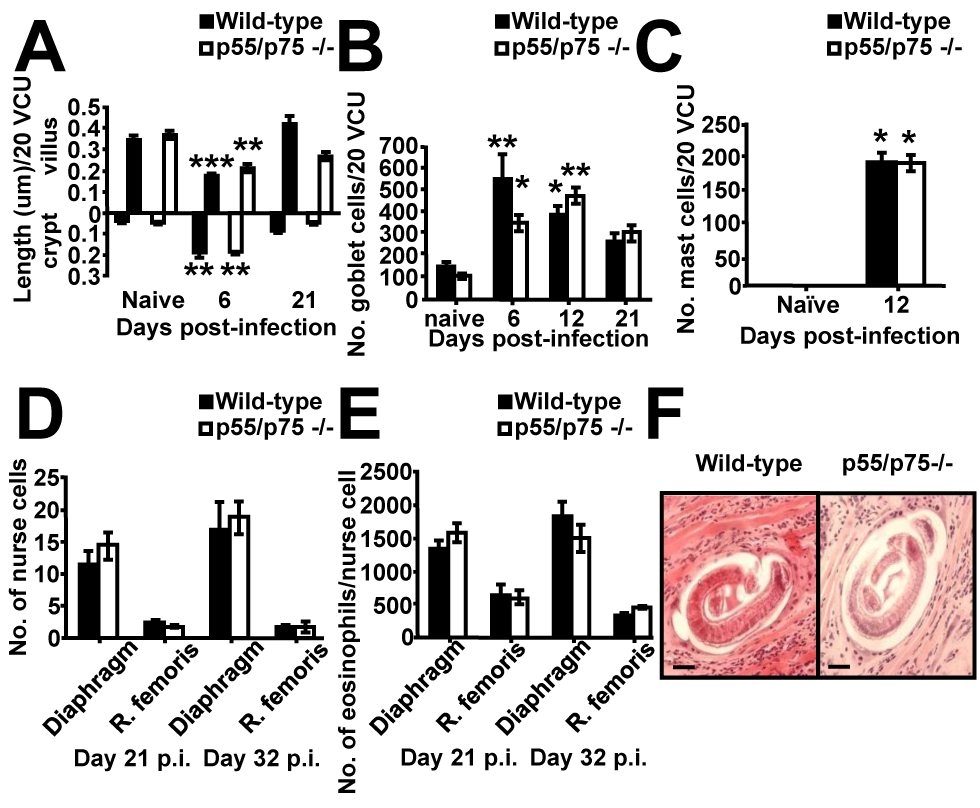

Supplement: Figure S3 — Enteropathy and nurse cell formation during T. spiralis infection is not altered in p55/p75−/− mice. (A) Comparison of crypt and villus length in wild-type and p55/p75−/− mice at naïve, 6 and 21 days p.i., quantified using Image J software. (B) Number of goblet and (C) mast cells/20 VCU in wild-type and p55/p75−/− mice in naïve, 6, 12 and 21 days p.i.; accessed via periodic acid-Schiff's and toludine blue histology staining respectively. (D) Number of nurse cells visible in field of view in the diaphragm and rectus femoris of infected mice at 21 and 32 days p.i. (E) Cellular infiltration of eosinophils of nurse cells in the diaphragm and rectus femoris of infected mice at days 21 and 32 p.i. (F) Representative haematoxylin and eosin stained images from (E) Black bar = 100 µm. A–D determined via haematoxylin and eosin histological staining. A–F values represent the means ± SE (n = 4) from 2 independent experiments. *, P<0.05; **, P<0.01 or ***, P<0.005 between naïve and infected groups, error bars represent SE of means. (TIF) [file ppat.1003122.s003.tif]
